# Evaluating the Therapeutic Potential of MRT68921 and Afatinib in Three-Dimensional Models of Epithelial Ovarian Cancer

**DOI:** 10.3390/cancers18020307

**Published:** 2026-01-19

**Authors:** Tiffany P. A. Johnston, Jack D. Webb, Matthew J. Borrelli, Emily J. Tomas, Áine C. Pucchio, Yudith Ramos Valdés, Trevor G. Shepherd

**Affiliations:** 1 The Mary and John Knight Translational Ovarian Cancer Research Unit, Verspeeten Family Cancer Centre, London, ON N6A 5W9, Canada; tjohn63@uwo.ca (T.P.A.J.); jwebb47@uwo.ca (J.D.W.); mborrel2@uwo.ca (M.J.B.); etomas3@uwo.ca (E.J.T.); apucchio2028@meds.uwo.ca (Á.C.P.);; 2 Department of Anatomy and Cell Biology, Schulich School of Medicine and Dentistry, Western University, London, ON N6A 5C1, Canada; 3 Department of Obstetrics and Gynecology, Schulich School of Medicine and Dentistry, Western University, London, ON N6A 5C1, Canada; 4 Department of Oncology, Schulich School of Medicine and Dentistry, Western University, London, ON N6A 5C1, Canada

**Keywords:** ovarian cancer, spheroids, organoids, ULK1, autophagy, MRT68921, afatinib

## Abstract

Background/Objectives: Epithelial ovarian cancer (EOC) is often diagnosed at advanced stages, with metastasis driven by spheroid dissemination within the peritoneal cavity. We previously demonstrated that autophagy supports spheroid cell survival and suggest that it contributes to chemoresistance. Unc-51-like autophagy activating kinase 1 (ULK1), a key regulator of autophagy, has emerged as a promising therapeutic target. Here, we evaluated the effects of ULK1 inhibition via MRT68921, alone and in combination with afatinib—a tyrosine kinase inhibitor (TKI) known to induce pro-survival autophagy—in EOC. Methods: High-grade serous (HGSOC) and ovarian clear cell carcinoma (OCCC) cell lines were cultured under adherent and spheroid conditions. Immunoblotting confirmed on-target effects and modulation of autophagy. Autophagic flux was assessed using mCherry-eGFP-LC3 reporter assays. We assessed 96 dose combinations of MRT68921 and afatinib using drug combination matrices, with synergy evaluated via Synergy Finder. Promising combinations were evaluated across multiple EOC spheroid models and patient ascites-derived organoids. Results: MRT68921 inhibited ULK1 activity and reduced autophagic flux in a context-dependent manner while afatinib alone induced autophagy. Their combination produced synergistic effects at select concentrations, impairing spheroid reattachment and viability. However, MRT68921 alone significantly reduced viability across multiple EOC models, including patient ascites-derived organoids. Conclusions: This study is the first to evaluate the combined effects of MRT68921 and afatinib in epithelial ovarian cancer. Our findings demonstrate that ULK1 inhibition via MRT68921 consistently reduces cell viability across multiple ovarian cancer models, supporting ULK1 as a promising therapeutic target. In contrast, combination with afatinib produced limited and context-dependent effects, indicating that further investigation is needed to identify optimal combination strategies for ULK1-targeted therapies.

## Simple Summary

Epithelial ovarian cancer (EOC) is typically diagnosed at an advanced stage, and treatment strategies have remained largely unchanged for decades. A key step in EOC metastasis involves the formation of spheroids which are clusters of cancer cells that disseminate throughout the peritoneal cavity. Cells within these spheroids rely on autophagy, an evolutionarily conserved survival mechanism that is regulated by a protein called unc-51-like autophagy activating kinase 1 (ULK1). In this study, we evaluated the pharmacological inhibition of ULK1 using MRT68921, both alone and in combination with afatinib, a tyrosine kinase inhibitor known to induce pro-survival autophagy. We found that MRT68921 significantly reduced cell viability across multiple EOC models, supporting ULK1 inhibition as a promising therapeutic approach. In contrast, combining MRT68921 with afatinib resulted in limited and context-dependent effects, highlighting the need to explore alternative combination strategies to enhance therapeutic efficacy.

## 1. Introduction

Epithelial ovarian cancer (EOC) ranks as the third most common gynecological cancer worldwide, bearing the highest mortality rate among such cancers in the developed world [1]. Many EOC patients are diagnosed at an advanced stage and chemoresistant disease is common. Both factors contribute to a five-year survival rate of roughly 50%, based on epidemiological data reported in the United States from 2014 to 2020 [2]. EOC comprises multiple histologies, with high-grade serous ovarian cancer (HGSOC) being the most common and lethal. Patients with HGSOC often respond initially to chemotherapy; however, recurrence with chemoresistant disease is common [3]. Ovarian clear cell carcinoma (OCCC) is a rarer histotype, accounting for approximately 10% of EOC cases, and is particularly difficult to treat due to poor responsiveness to conventional platinum-based chemotherapy [4,5]. Notably, effective strategies for treating chemoresistance for both histotypes disease remain elusive, underscoring the importance of investigating novel therapeutic strategies to improve patient outcomes.

A common feature of advanced EOC is the accumulation of fluid in the peritoneal cavity known as malignant ascites [6]. Within the ascites fluid, multicellular aggregates of EOC cells, known as spheroids, are present and thought to contribute to metastasis [7,8,9]. Their enhanced adhesive and invasive capacities allow them to attach to distant sites and form secondary lesions on many surfaces throughout the peritoneal cavity. Many studies in cell culture models have shown that EOC spheroids undergo adaptive metabolic reprogramming that contributes to disease progression and chemoresistance [10,11]. Our group has shown that compared to adherent cells, EOC spheroids exhibit a dormant phenotype driven by cellular quiescence, activated stress metabolism, and macroautophagy activation [12,13,14,15,16,17]. Importantly, these mechanisms of dormancy may contribute to the therapeutic challenges of treating spheroids, as chemotherapy is typically more effective against highly proliferative cells.

Macroautophagy (herein referred to as autophagy) is an evolutionarily conserved process that promotes cell survival under starvation-like conditions through the degradation and recycling of intracellular components [18]. Cancer cells often exploit autophagy to survive nutrient-deprived and hypoxic conditions, thus potentiating disease progression. We found that EOC spheroids potently activate autophagy, which in our system is modulated through calcium/calmodulin-dependent protein kinase kinase 2 (CAMKK2)—AMP-activated protein kinase (AMPK) activation and downregulation of protein kinase B (AKT) signalling [12,15]. Further, we have shown that autophagy activation is required to maintain EOC spheroid integrity and viability in suspension [16]. Beyond EOC, autophagy has been implicated as a key mechanism promoting tumour cell dormancy during metastasis and chemotherapy in various cancer types [18,19] prompting us to further investigate its role and therapeutic potential in EOC.

Autophagy activation is regulated by the autophagy initiation complex (AIC), which comprises FAK family-interacting protein of 200 kDa (FIP200), autophagy-related protein 13 homolog (ATG13), autophagy-related protein 101 (ATG101), and unc-51-like autophagy activating kinase 1 (ULK1) or its homologue ULK2 [20]. Despite similarities between ULK1 and ULK2, studies have found that ULK1 is the dominant kinase regulating autophagy [20,21]. ULK1 is a serine-threonine kinase that acts as the enzymatic component of the AIC. Canonically, ULK1 is activated via AMPK phosphorylation under nutrient deprivation [22]. Conversely, ULK1 is inhibited by mechanistic target of rapamycin complex 1 (mTORC1) phosphorylation under nutrient-rich conditions. Our group has shown that HGSOC spheroids have increased ULK1 protein expression correlating with increased autophagy activation [16]. Given ULK1 is a crucial regulator of autophagy, we have investigated its therapeutic potential using MRT68921, a competitive ATP-binding site inhibitor shown to block ULK1 and ULK2 in vitro kinase activity in the low nanomolar range [23]. We have validated that MRT68921 reduces ULK1 kinase activity and autophagic flux in HGSOC spheroids and robustly decreases HGSOC spheroid viability [16]. These results indicate that ULK1 inhibition has promising therapeutic potential that warrants further investigation in the broader context of EOC histotypes while exploring potential combination with other anticancer agents.

The PI3K-AKT-mTOR pathway is crucial in regulating many cellular functions including cell growth, survival, and metabolism [24]. In both normal and cancer cells, this pathway is intimately linked to autophagy since ULK1 is negatively regulated through phosphorylation by mTORC1 leading to autophagy suppression [20]. In many cancers, including HGSOC and OCCC histotypes of EOC, mutations or amplifications in components of the PI3K-AKT-mTOR pathway result in constitutive AKT activation [24]. Several early-phase clinical trials have evaluated PI3K-AKT-mTOR pathway inhibitors in EOC; however, none have advanced to late-phase clinical trials [25].

Afatinib (BIBW2992) is a second-generation tyrosine kinase inhibitor (TKI) that acts to irreversibly inhibit the ErbB family including the epidermal growth factor receptor (EGFR) [26,27]. Notably, these receptors act upstream of the PI3K-AKT-mTOR pathway, and their inhibition inactivates the pathway while indirectly promoting autophagy by relieving mTORC1-mediated suppression of ULK1 [20,26,28,29]. Given the links between afatinib, the PI3K-AKT-mTOR pathway, and autophagy, studies have explored the combination of afatinib with autophagy inhibitors. In pancreatic ductal adenocarcinoma (PDAC), non-small cell lung cancer (NSCLC), and head and neck squamous cell carcinoma (HNSCC), it has been demonstrated that afatinib anti-cancer effects are enhanced when combined with autophagy inhibition [29,30,31,32]. However, little work has been performed exploring TKIs and this phenomenon in EOC.

In this study, we demonstrate that MRT68921 successfully inhibits ULK1 and autophagy in multiple cell lines representing two aggressive EOC histotypes, HGSOC and OCCC, leading to reduced cell viability. Consistent with findings in other cancers, afatinib induced autophagy in these HGSOC and OCCC cell lines. We evaluated the effect of combining afatinib and MRT68921, identifying potential synergistic combinations through drug-dose combination matrices. Combination treatment impaired spheroid reattachment and viability, however these effects were primarily driven by ULK1 inhibition. These findings were corroborated in HGSOC patient ascites-derived organoids, where variability among samples offers preliminary insight into this treatment strategy in the context of interpatient tumour heterogeneity. Together, these results suggest that ULK1 inhibition represents a promising therapeutic approach in EOC and motivate exploration of how ULK1-targeted strategies may interact with tyrosine kinase inhibition.

## 2. Materials and Methods

### 2.1. Cell Culture

OVCAR8, HeyA8, mCherry-eGFP-LC3B OVCAR8, and mCherry-eGFP-LC3B HeyA8 cells were cultured in Roswell Park Memorial Institute medium (RPMI)-1640 (#350-700 CL, Wisent (Saint-Jean-Baptiste, QC, Canada)) supplemented with 10% fetal bovine serum (FBS). iOvCa195, iOvCa198, iOvCa256, TOV-21G, ES2, OVCAR3, OVCAR4, COV318, COV362, 105C, and OV207 cells were cultured in Dulbecco’s modified Eagle medium (DMEM/F12 (#11320033, Thermo Fisher Scientific, Waltham, MA, USA) supplemented with 10% FBS. iOvCa195, iOvCa198, and iOvCa256 ascites-derived cell lines were generated and established by Dr. Gabriel DiMattia using culture methods as previously described [33]. OVCAR8 and HeyA8 cell lines were obtained from the American Type Culture Collection (ATCC; Manassas, VA, USA); TOV-21G cell line was provided by Dr. Anne-Marie Mes-Masson (University of Montreal, Montreal, QC, Canada); ES2 cell line was provided by Dr. Barbara Vanderhyden (University of Ottawa, Ottawa, ON, Canada); the OVCAR3 and OVCAR4 cell lines were provided by Dr. James Koropatnick (Western University, London, ON, Canada); the COV318 and COV362 cell lines were provided by Dr. Zia Khan (Western University); the 105C cell line was obtained from Dr. Hal Hirte (McMaster University, Hamilton, ON, Canada) and further characterized by us [33]. Cells in adherent culture were maintained on tissue culture-treated polystyrene (Sarstedt, Newton, NC, USA). Cells in spheroid culture were maintained in Ultra-Low Attachment (ULA; Corning, NY, USA) plates to allow spontaneous spheroid formation via aggregation. Cell lines were authenticated using short tandem repeat (STR) analysis performed at the Centre for Applied Genomics (TCGA; The Hospital for Sick Children, Toronto, ON, Canada) and confirmed negative for mycoplasma (ATCC, 30-1012K). All cells were maintained under standard conditions at 37 °C with 5% CO_2_.

### 2.2. Antibodies and Reagents

Antibodies against p-Beclin1 Ser30 (#54101; 1:1000), Beclin1 (#3738; 1:1000), ULK1 (#8054; 1:1000), LC3 (#2775; 1:1000), SQSTM1/p62 (#5114; 1:1000), p-AKT Ser473 (#9271; 1:1000), AKT (#9272; 1:1000), p-mTOR Ser2448 (#2971; 1:1000), mTOR (#2983; 1:1000), p-p70S6K Thr389 (#9234; 1:1000), p70S6K (#2708; 1:1000), EGFR (#2232; 1:1000) and, cleaved PARP (#9541; 1:1000) were purchased from Cell Signalling Technology (Danvers, MA, USA). Antibodies against actin (A2066; 1:10,000) and vinculin (V9264; 1:10,000) were purchased from MilliporeSigma (Burlington, MA, USA). Horseradish peroxidase (HRP)-conjugated antibodies against rabbit IgG (NA934; 1:10,000) and mouse IgG (NA931; 1:10,000) were purchased from Cytiva (Malborough, MA, USA). All antibodies were diluted in tris-buffered saline-Tween 20 (TBST) containing 5% bovine serum albumin (BSA). alamarBlue cell viability reagent (DAL1025; 1:100) and Trypan Blue Solution (15250061) were purchased from Thermo Fisher Scientific. MRT68921 (5 mM in DMSO) was purchased from Selleckchem (#S7949, Houston, TX, USA) and stored at −20 °C. Afatinib (5 mM in DMSO) was purchased from Tocris (#6812, Bristol, UK) and stored at −20 °C. Paclitaxel (5 mM in DMSO) was purchased from Cayman Chemical Company (#10461, Ann Arbor, MI, USA) and stored at −20 °C. Carboplatin (27 mM in saline) was received from the Verspeeten Family Cancer Centre (London, ON, Canada) and stored at 4 °C.

### 2.3. Generation of mCherry-eGFP-LC3B Cell Lines

Lipofectamine (#18324012, Thermo Fisher Scientific)-mediated transfection was performed on OVCAR8 and HeyA8 cell lines, enabling transfection of the mCherry-eGFP-LC3B reporter (autoR) plasmid (#22418, Addgene, Watertown, MA, USA). To select cells with successful plasmid integration, cells were grown in complete medium containing G-418 (#10131035, Thermo Fisher Scientific) for two weeks. Following selection, cells were grown in complete medium without G-418 for an additional four weeks. Double-positive cells (GFP^+^/mCherry^+^) were sorted using fluorescence-activated cell sorting (FACS) at the Robarts Research Institute, Western University.

### 2.4. Fluorescent Image Analysis

Time course fluorescence images of OVCAR8-autoR and HeyA8-autoR spheroids (mCherry and eGFP channels) were captured using an IncuCyte S3 live cell analysis instrument (Sartorius, Oakville, ON, Canada) using exposure times of 400 ms and 300 ms, respectively. Analyses of mCherry/EGFP fluorescence ratio were then conducted using a modified version of Spatial Profiling of Ratiometric Trends in Spheroids (SPoRTS), the automated ratiometric image analysis platform we published recently [34]. Briefly, this version of SPoRTS maintains the same frameworks for image processing, thresholding, and data aggregation as the original, but the spatial component has been removed. Modified SPoRTS was used to calculate mCherry/eGFP ratios based on total spheroid fluorescence from the respective channels (i.e., total mCherry fluorescence divided by total eGFP fluorescence). The modified version of SPoRTS used in this study is available alongside the original at https://github.com/mjborrelli/SPoRTS (accessed on 30 January 2025). 

### 2.5. Preparation of Whole-Cell Protein Lysates

Adherent cells were seeded at a density of 700,000 cells per 60 mm dish, while spheroids were seeded at a density of 500,000 cells per well in a 6-well ULA dish. To collect adherent cell lysates, the medium was aspirated, and cells were washed with 5 mL of cold phosphate-buffered saline (PBS) before being scraped into 100 μL of modified radioimmunoprecipitation assay (RIPA) buffer [50 mM HEPES (pH 7.4), 150 mM NaCl, 10% glycerol, 1.5 mM MgCl_2_, 1 mM EGTA, 1% Triton X-100, 0.1% sodium dodecyl sulfate (SDS), 1% sodium deoxycholate, 10 mM sodium pyrophosphate, 10 mM sodium fluoride, 1 mM sodium orthovanadate, 1× SIGMAFAST protease inhibitor cocktail (#S8820; MilliporeSigma), 10 mM β-glycerophosphate, and 1 mM phenylmethylsulfonyl fluoride (PMSF)]. Spheroids were collected by transferring the cell suspension from the wells of the 6-well ULA plate to a conical tube kept on ice. The tubes were centrifuged at 600× *g* for 5 min at 4 °C, the medium was aspirated, and cell pellets were washed with 10 mL of ice-cold PBS. The centrifugation and aspiration steps were repeated, and the final cell pellets were resuspended in 100 μL of modified RIPA buffer. All cell lysates were subjected to one freeze–thaw cycle and then clarified by centrifugation at 21,100× *g* for 20 min at 4 °C. Protein concentrations in the supernatants were determined using the Bradford Protein Assay Dye Reagent (#5000006, Bio-Rad, Hercules, CA, USA).

### 2.6. Immunoblot Analysis

Immunoblotting was performed using the Bio-Rad Mini-PROTEAN II Electrophoresis System following the manufacturer’s instructions. Protein samples (30 μg) were loaded onto 8% or 12% SDS-polyacrylamide gels and subjected to electrophoresis. Proteins were subsequently transferred to polyvinylidene fluoride (PVDF) membranes (88518, Thermo Fisher Scientific). Membrane blocking and washing steps were carried out in TBST containing either 5% BSA or 5% skim milk, depending on the antibody requirements. Membranes were incubated with primary antibodies overnight at 4 °C. Secondary antibodies, diluted in 5% BSA, were applied for 1 h at room temperature. Enhanced chemiluminescence detection was performed using the Luminata Forte HRP substrate (#WBLUF0100, MilliporeSigma), and the resulting chemiluminescence was captured using the Bio-Rad ChemiDoc system. Densitometry analysis was conducted with Bio-Rad Image Lab software version 6.0.1.

### 2.7. alamarBlue Viability Assay and IC_50_ Curve Generation

Cells were seeded in 96-well dishes at a density of 2000 cells/100 μL in each well. Adherent cells were treated 24 h after seeding, early spheroids were treated at the time of seeding and late spheroids were treated 72 h after seeding. Under all conditions, cells were treated with 12 concentrations of MRT68921 ranging from 0.01 to 100 μM. After 72 h of treatment, 100 μL of diluted alamarBlue reagent (1:10 in media) was added to each well. Absorbance was measured at 570 and 600 nm using the BioTek Synergy H1 plate reader (Agilent Technologies, Mississauga, ON, Canada) after 4 h of incubation with alamarBlue reagent for adherent culture and after 24 h for spheroid culture. To determine the half-maximal inhibitory concentration (IC_50_) value of MRT68921 in HGSOC and OCCC cell lines, the alamarBlue absorbance reading of each concentration was normalized to the reading of DMSO control, represented by 100% viability. The normalized data was analyzed in GraphPad Prism 9.5.1 using the non-linear regression (curve fit) analysis tool.

### 2.8. Synergy Finder Analysis

Cells were seeded at a density of 2000 cells/100 μL in each well of 96-well dishes. Both adherent and spheroid cultures were treated 24 h after seeding as a matrix array into a 96-well cluster plates encompassing 96 possible combinations of MRT68921 and/or afatinib, including each drug alone or in combination. After 72 h of treatment, cells were incubated with 100 μL of diluted alamarBlue reagent (1:10 in media). The same protocol was followed as above to generate IC_50_ curves. Normalized data was organized into table format as outlined by the Synergy Finder User Manual available at https://synergyfinder.aittokallio.group/synfin_docs/ (accessed on 15 October 2025). The table was uploaded to the Synergy Finder website for analysis using the Bliss Synergy Score (BSS) model [35].

### 2.9. Trypan Blue Exclusion Cell Counting

Spheroids were seeded into 24-well ULA plates at a density of 25,000 cells/mL (OVCAR8, HeyA8, and ES2) or 50,000 cells/mL (TOV-21G). After 24 h, spheroids were treated with either DMSO, 0.25 μM MRT68921, 4 μM MRT68921, 0.125 μM afatinib, 0.25 μM MRT68921 + 0.125 μM afatinib, or 4 μM MRT68921 + 0.125 μM afatinib. After 72 h of treatment, spheroids were collected into microcentrifuge tubes and pelleted by centrifugation (500× *g* for 6 min). The medium was aspirated, and the pellet was washed in 500 μL of PBS before being pelleted by centrifugation. PBS was aspirated and pellets were incubated at 37 °C for 30 min in 50 μL of Trypsin/EDTA. Before counting, 50 μL of FBS was added to stop trypsin-mediated digestion. Subsequently, 50 μL of Trypan Blue dye was added and gently mixed by pipetting. Cell counting was performed using a TC10 Automated Cell Counter (Bio-Rad).

### 2.10. Spheroids Reattachment Assay

Cells were seeded in 24-well ULA plates at a density of 25,000 cells in 1 mL per well (OVCAR8 and HeyA8) or 50,000 cells in 1 mL per well (TOV-21G and ES2). After 24 h, cells were treated for 72 h with either DMSO, 4 μM MRT68921, 0.125 μM afatinib, or 4 μM MRT68921 + 0.125 μM afatinib. Spheroids were then transferred to 24-well tissue culture-treated polystyrene plates to allow attachment. Once spheroids had visibly adhered to the dish they were then fixed and stained with Hema3 (# 23-123869, ThermoFisher Scientific).

### 2.11. Organoid Culture

HGSOC patient-derived iOvCa cell lines were seeded at a density of 5000 cells/well and resuspended in 10 mL of Cultrex Basement Membrane Extract (BME) PathClear Type 2 (#3532-010-02, R & D Systems, Minneapolis, MN, USA) as droplets on 96-well tissue culture-treated polystyrene plates. The droplets were overlaid with Advanced DMEM/F-12 (#12634010, Thermo Fisher Scientific) supplemented with additional media components as outlined in Table 1. Organoids were treated 72 h after seeding with either DMSO, 0.25 μM MRT68921, 4 μM MRT68921, 0.125 μM afatinib, 0.25 μM MRT68921 + 0.125 μM afatinib, or 4 μM MRT68921 + 0.125 μM afatinib. After 72 h of treatment, organoids were incubated with 100 μL of diluted alamarBlue reagent (1:10 in media). Absorbance was measured at 570 and 600 nm using the BioTek Synergy H1 plate reader (Agilent Technologies) after 24 h.

### 2.12. Microscopy

Phase contrast images of early and late spheroids treated with MRT68921 and fluorescent images of OVCAR8- and HeyA8 autoR spheroids treated with MRT68921 were captured using the Incuctye S3 Live-Cell Analysis System (Sartorius). The fluorescent images were captured at acquisition times of 300 ms and 400 ms for the green and red channels, respectively. Images of Hema3 stained reattached spheroids were captured using the Axio Zoom V16 microscope (Zeiss, Oberkochen, Germany).

### 2.13. Statistical Analysis

Statistical analyses were performed using GraphPad Prism 9.0.1 (GraphPad Software). Statistical tests performed include one-way and two-way ANOVA followed by Tukey’s multiple comparisons test and unpaired *t*-tests. Specific analysis details for each experiment are described in the respective figure legends.

## 3. Results

### 3.1. MRT68921 Inhibits ULK1 Activity in EOC Cell Lines

To date, our group remains the first and only to investigate MRT68921 in the context of EOC [16]. Previously, we demonstrated that MRT68921 inhibits ULK1 kinase activity in HGSOC spheroids but not in FT190 EOC precursor spheroids. Here, we sought to determine whether MRT68921 similarly suppresses ULK1 kinase activity in OCCC and additional HGSOC cell lines. Given that ULK1 expression and autophagy activation are upregulated in EOC spheroids compared to adherent culture, we also investigated whether MRT68921 differentially affects ULK1 activity between adherent and spheroid cultures [12,15,16].

**Figure 1:**
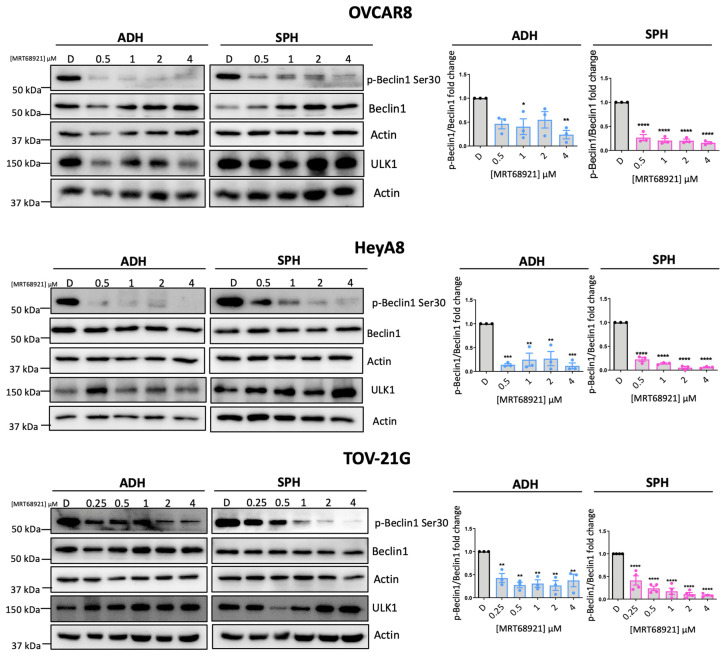
MRT68921 inhibits ULK1 activity in EOC cell lines. Representative immunoblots and corresponding densitometric analyses of OVCAR8 (n = 3), HeyA8 (n = 3), and TOV-21G (n = 4) cells treated with the indicated concentrations of MRT68921 under adherent (ADH) and spheroid (SPH) culture conditions. Cells were seeded 24 h prior to treatment and harvested after 24 h of drug exposure. Densitometric values are presented as mean fold-change relative to DMSO-treated controls (D) ± SEM. Black, blue, and pink outlines denote DMSO-treated control, ADH-treated, and spheroid-treated conditions, respectively. Statistical analysis was performed using one-way ANOVA followed by Tukey’s post hoc test (* *p* < 0.05, ** *p* < 0.01, *** *p* < 0.001, **** *p* < 0.0001).

To this end, we treated EOC cell lines representing HGSOC (OVCAR8), OCCC (TOV-21G), and the poorly differentiated HeyA8 line under adherent and spheroid conditions with MRT68921. ULK1 kinase activity was assessed by measuring Beclin-1 phosphorylation at Ser30 (p-Beclin1) [36,37,38] via Western blotting (Figure 1). Cell lines were treated with concentrations of MRT68921 that were previously shown to block ULK1 and autophagy under similar conditions [16].

In OVCAR8 spheroids, MRT68921 markedly reduced p-Beclin1 levels across all tested concentrations. However, under adherent conditions, a significant reduction in p-Beclin1 was observed only at 1 µM and 4 µM. In contrast, both HeyA8 and TOV-21G cells exhibited a consistent reduction in p-Beclin1 at all concentrations under both adherent and spheroid conditions. These findings confirm that MRT68921 effectively inhibits ULK1 activity at low micromolar concentrations across EOC histotypes, independent of culture conditions. We also confirmed that a single dose of 1 μM MRT68921 maintains ULK1 inhibition over a 96 h treatment period (Supplementary Figure S1).

### 3.2. Variable Potency of MRT68921 on Autophagy Inhibition in EOC Spheroids

**Figure 2:**
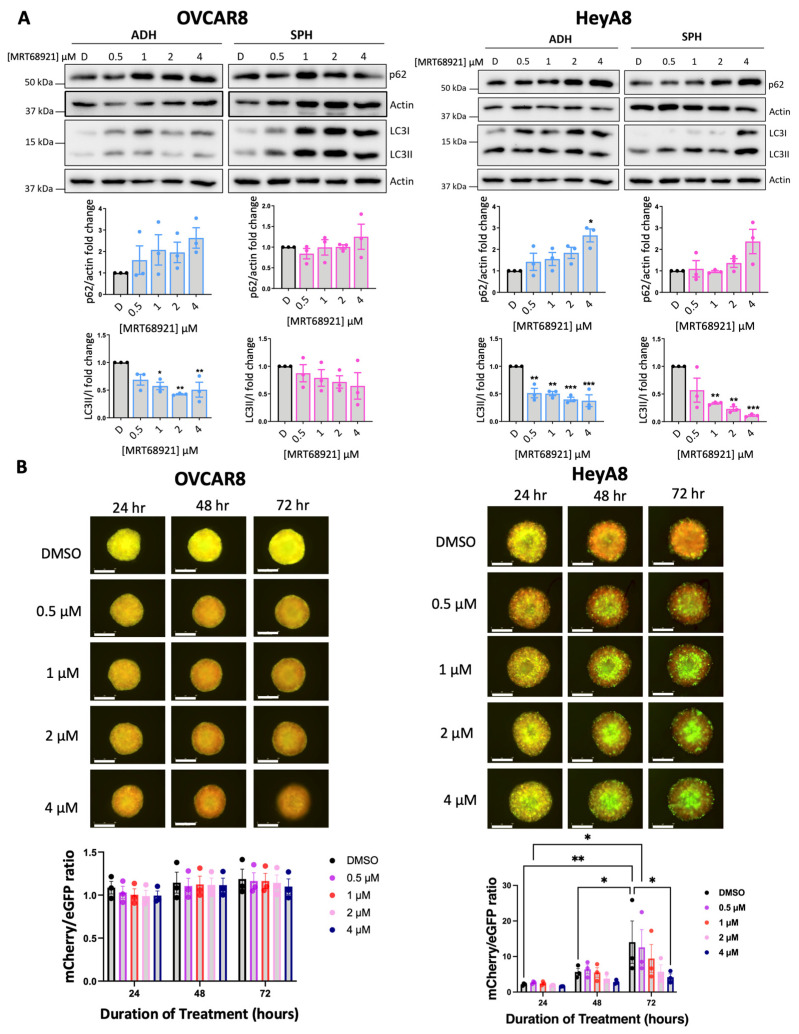
MRT68921 impairs autophagy in EOC cell lines. (**A**) Representative immunoblots and corresponding densitometric analyses of autophagy markers in OVCAR8 and HeyA8 cells (n = 3) treated with the indicated concentrations of MRT68921 under adherent (ADH) and spheroid (SPH) culture conditions. Cells were seeded 24 h prior to 24 h drug treatment. Densitometric values are expressed as mean fold change relative to DMSO-treated control (D) ± SEM. Black, blue, and pink outlines denote DMSO-treated control, ADH-treated, and spheroid-treated conditions, respectively. Statistical significance was assessed using one-way ANOVA with Tukey’s post hoc test (* *p* < 0.05, ** *p* < 0.01, *** *p* < 0.001). (**B**) Representative fluorescence images of OVCAR8-autoR and HeyA8-autoR spheroids treated with MRT68921 at the indicated concentrations. Spheroids were treated 24 h post-seeding, and images were acquired at the indicated time points; time stamp reflects the duration of drug exposure. Autophagic flux was quantified based on the mCherry/eGFP fluorescence ratio and is presented as mean ± SEM (n = 3). Black, purple, red, pink, and blue outlines denote DMSO-, 0.5 μM-, 1 μM-, 2 μM-, 4 μM-treated respectively. Statistical analysis was performed using two-way ANOVA followed by Tukey’s multiple comparisons test (* *p* < 0.05, ** *p* < 0.01). Scale bars represent 400 μm.

Given ULK1’s canonical role as a key regulator of autophagy, we evaluated the impact of ULK1 inhibition via MRT68921 on autophagic activity. To this end, we assessed microtubule-associated protein 1 light chain 3 (LC3) processing (LC3II/I ratio) and sequestosome 1 (p62) expression as established markers of autophagy [39,40] via immunoblotting (Figure 2A). In HeyA8 cells, MRT68921 treatment reduced the LC3II/I ratio under both adherent and spheroid conditions. Similarly, in OVCAR8 adherent cells, MRT68921 treatment decreased the LC3II/I ratio; however, in OVCAR8 spheroids, no reduction in LC3II/I ratio was observed. In support of autophagy suppression, p62 levels increased in HeyA8 adherent cells treated with 4 µM MRT68921. Notably, p62 expression remained unchanged in OVCAR8 cells, further highlighting cell line–specific differences in the autophagic response to ULK1 inhibition via MRT68921.

To further explore the dose-dependent and temporal effects of MRT68921 on autophagic flux in live spheroids, we utilized OVCAR8 and HeyA8 cell lines transfected with the mCherry-eGFP-LC3B reporter (autoR; Figure 2B). This reporter exploits the differential stability of mCherry and eGFP fluorescent proteins in the lysosome, where mCherry remains stable while eGFP is degraded, allowing for quantification of mCherry/eGFP fluorescence ratios as an indication of autophagic flux [40,41]. Consistent with the lack of LC3II/I and p62 changes in MRT68921-treated OVCAR8-autoR spheroids, the mCherry/eGFP ratio remained unchanged over 72 h of MRT68921 treatment at all tested concentrations. However, in HeyA8-autoR spheroids, treatment with 4 µM MRT68921 resulted in a significant reduction in autophagic flux by 72 h. As expected, DMSO-treated control spheroids exhibited a progressive increase in autophagic flux at 48 and 72 h. These results support a dose-dependent suppression of autophagy by MRT68921 in this model, yet its effects may vary by cell line, culture conditions, and drug concentration.

### 3.3. EOC Spheroids Exhibit Reduced Sensitivity to MRT68921 Relative to Adherent Cells

Since we observed differences in autophagy modulation among EOC cell lines in response to MRT68921, we expanded the number of cell lines representing HGSOC and OCCC histotypes and determined sensitivity on cell viability by generating dose–response curves and calculating IC_50_ values (Supplementary Table S1). Among the ten cell lines assessed in adherent culture, IC_50_ values ranged from 0.760 to 4.242 µM, with most cell lines exhibiting sensitivities within a narrow range. Notably, TOV-21G and 105C, two OCCC cell lines, displayed the greatest sensitivity to MRT68921, with IC_50_ values of 0.760 µM and 1.285 µM, respectively.

**Figure 3:**
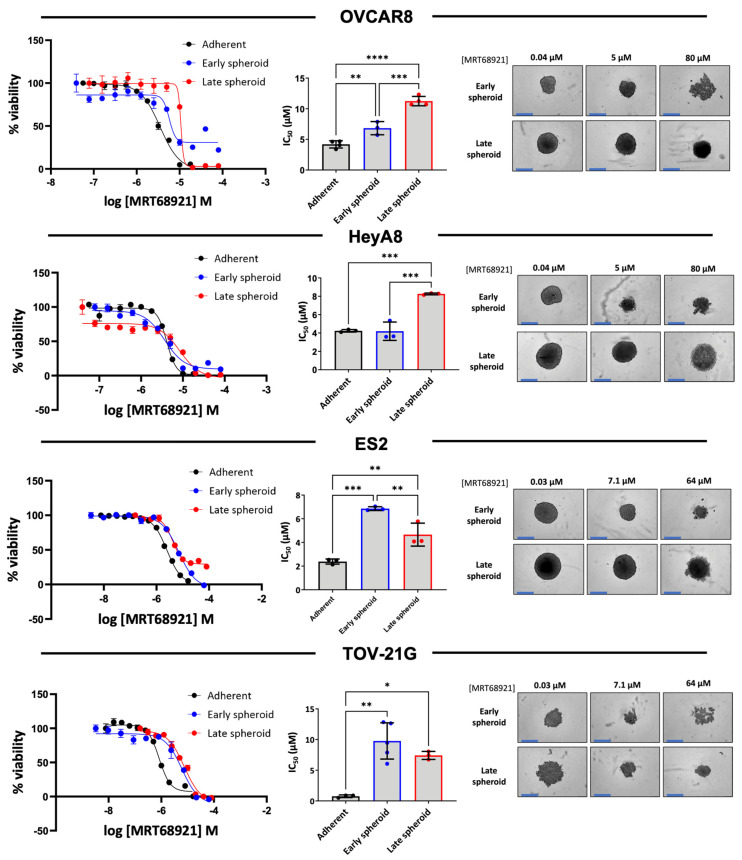
EOC cell lines are more sensitive to MRT68921 under adherent conditions compared to spheroids. Representative dose–response curves for MRT68921 OVCAR8 (n = 3), HeyA8 (n = 3), and TOV-21G (n = 4) cell lines cultured under adherent (black), early spheroid (blue), and late spheroid (red) conditions. Adherent cells were treated 24 h post-seeding. Early spheroids were treated at the time of seeding, whereas late spheroids were treated 72 h after seeding. In all conditions, treatment was maintained for 72 h, followed by alamarBlue viability assays. Absorbance readings were used as an indirect measure of cell viability and normalized to DMSO-treated controls to calculate percent viability. Bar graphs display mean IC_50_ values ± SEM for each cell line across culture conditions; statistical significance was determined using one-way ANOVA followed by Tukey’s multiple comparisons test (* *p* < 0.05, ** *p* < 0.01, *** *p* < 0.001, **** *p* < 0.0001). Representative phase-contrast images are shown for spheroids treated with low, medium, and high concentrations of MRT68921. Scale bar = 400 μm.

Given that EOC spheroids activate autophagy to promote cell survival in an ULK1-dependent manner [14,16], we next assessed the impact of MRT68921 on spheroid viability, comparing sensitivity during early spheroid formation and in established spheroids of OVCAR8, HeyA8, TOV-21G and ES2 cell lines. Dose–response curves were generated for spheroids treated at the time of seeding (early spheroids) and spheroids treated 72 h post-seeding (late spheroids) as compared with adherent culture (Figure 3).

In OVCAR8, ES2, and TOV-21G cell lines, adherent IC_50_ values were significantly lower than those observed for early or late spheroids, indicating reduced sensitivity in spheroid culture conditions. OVCAR8 early spheroids exhibited greater sensitivity than late spheroids. In contrast, ES2 spheroids displayed the opposite trend, with late spheroids exhibiting greater sensitivity compared to early spheroids. In TOV21G spheroids, no difference between early and late spheroids was observed, yet both possessed significantly increased IC_50_ values than adherent cells. HeyA8 spheroids exhibited comparable IC_50_ values between adherent and early spheroid conditions, however late spheroids displayed reduced sensitivity to MRT68921.

### 3.4. Afatinib Induces Autophagy in EOC Cell Lines

To further enhance the sensitivity of EOC spheroids to MRT68921 given its potential limited efficacy as a single agent, we explored rational combination strategies. Our previous work demonstrated that AKT inhibition (Akti-1/2) synergizes with autophagy inhibitors (chloroquine and Spautin-1) [42]. Building on these findings, we hypothesized that afatinib, a TKI that acts upstream of the PI3K-AKT-mTOR pathway, could be a promising candidate. Inhibition of this pathway has been shown to relieve mTOR-mediated repression of ULK1, thereby promoting autophagy initiation [24,28,43]. Furthermore, afatinib has been reported to induce autophagy as a pro-survival mechanism in various cancer models, often contributing to resistance against TKIs [30,31,32]. Accordingly, we sought to explore afatinib in EOC, given that its role in this context remains poorly understood.

**Figure 4:**
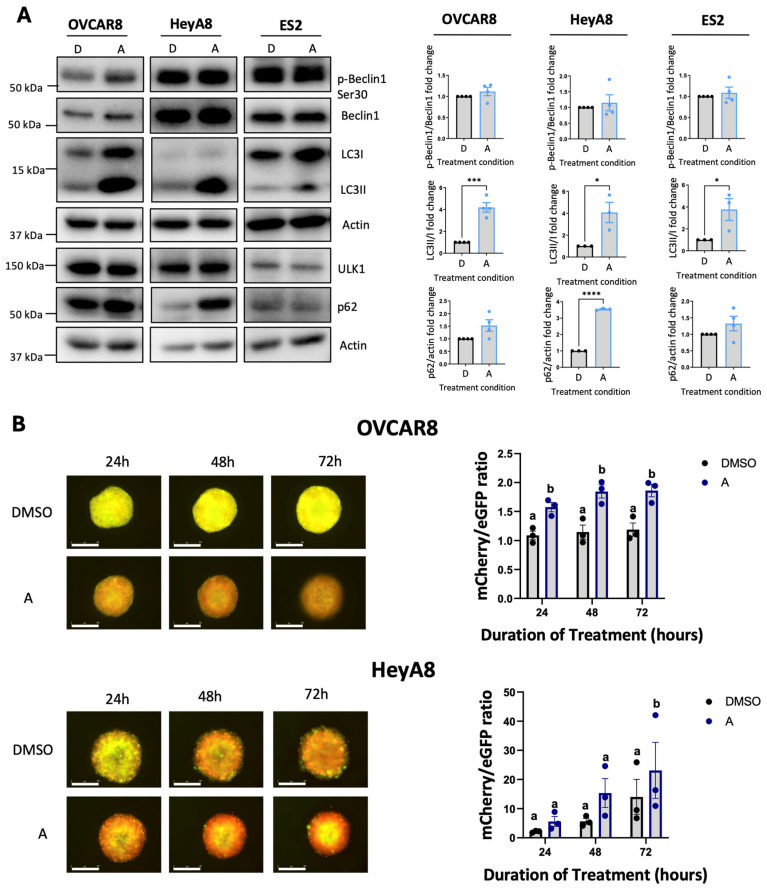
Afatinib induces autophagy in EOC cell lines. (**A**) Representative immunoblots and densitometric analyses of HeyA8, OVCAR8, and ES2 cells treated with 4 μM afatinib for 24 h under adherent culture conditions. Cells were seeded and allowed to adhere for 24 h prior to treatment. Densitometric values are presented as mean fold change relative to DMSO-treated controls ± SEM (n = 4). Black, and blue outlines denote DMSO-treated and afatinib-treated respectively. Statistical significance was assessed using unpaired *t*-tests (* *p* < 0.05, *** *p* < 0.001, **** *p* < 0.0001). (**B**) Representative fluorescence images of OVCAR8- and HeyA8-autoR spheroids treated with 4 μM afatinib, starting 24 h post-seeding. Timepoints indicate the duration of treatment. Autophagic flux was assessed using the mCherry/eGFP ratio and is presented as mean ± SEM (n = 3). Black, and blue outlines denote DMSO-treated and afatinib-treated respectively. Statistical analysis performed by one-way ANOVA with Tukey’s multiple comparisons test. Letter labels above bars indicate the presence (different letters) or absence (same letter) of significance at *p* < 0.05. Scale bars represent 400 μm.

Firstly, we confirmed that afatinib modulates PI3K-AKT-mTOR signalling in EOC cell lines via immunoblotting (Supplemental Figure S2). To investigate whether this modulation resulted in autophagy induction in EOC cell lines, we performed immunoblotting on lysates collected from adherent cells treated with 4 μM afatinib (Figure 4A). Across the three cell lines we examined, OVCAR8, HeyA8 and ES2, afatinib treatment resulted in a robust increase in the LC3II/I ratio, a hallmark of autophagosome formation, despite no significant changes in the ULK1 substrate p-Beclin1 Ser30.

To assess whether afatinib treatment enhances autophagic flux, we quantified fluorescence of OVCAR8- and HeyA8-autoR spheroids treated with afatinib (Figure 4B). In OVCAR8-autoR spheroids, 4 μM of afatinib significantly increased autophagic flux at all time points, whereas HeyA8-autoR spheroids displayed increased autophagic flux after 72 h of treatment.

### 3.5. Distinct Regions of Synergy Are Observed in MRT68921 and Afatinb Combination Analysis

Previous studies have demonstrated that autophagy suppression enhances afatinib-induced cytotoxicity across multiple cancer models [29,30,31,32]. Based on these findings and our results showing that afatinib induces autophagy in EOC cell lines, we hypothesized that ULK1 and autophagy inhibition via MRT68921 may similarly enhance afatinib efficacy in EOC cell lines.

To test this, we performed drug combination matrix assays, evaluating 96 unique concentration combinations of MRT68921 and afatinib in OVCAR8, HeyA8, ES2, and TOV-21G spheroids. Using Synergy Finder, we calculated the Bliss Synergy Score (BSS) for multi-dose and multi-drug combination response data [35,44].

**Figure 5:**
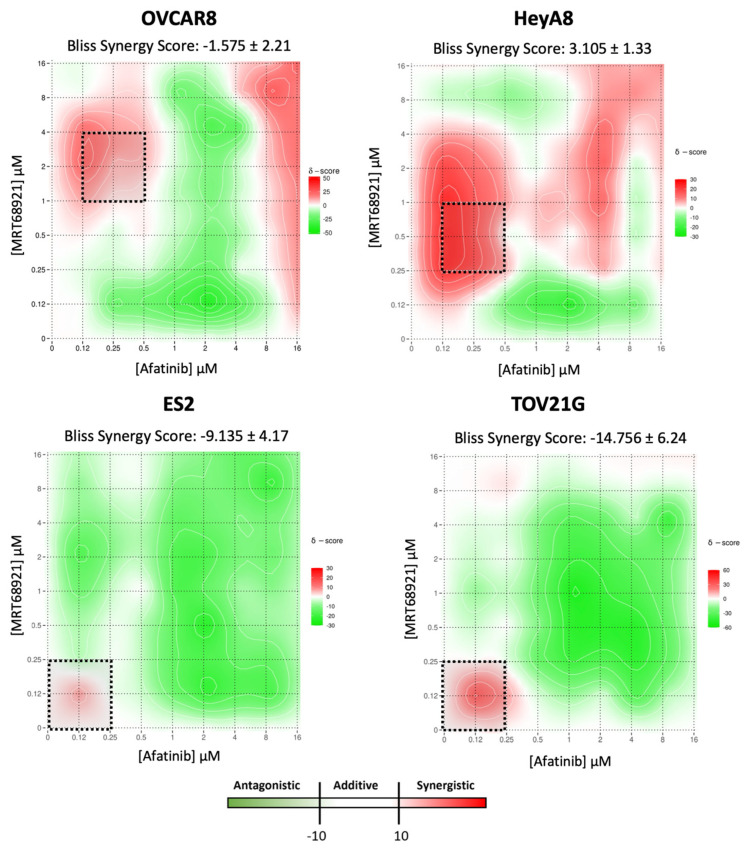
SynergyFinder analysis of MRT68921 and afatinib combination treatment in EOC spheroids. Bliss synergy scores (BSS) are presented as mean ± SEM (n = 3). Heat maps display the average synergy scores from biological replicates, where red indicates synergistic interaction (BSS > 10), white represents additive effects (−10 < BSS < 10), and green indicates antagonism (BSS < −10). Regions enclosed by dashed-lined boxes represent areas with the greatest degree of synergy. All spheroids were seeded 24 h prior to 72 h combination treatment with afatinib and MRT68921, using a matrix of concentrations ranging from 0 to 16 μM. Cell viability was assessed using alamarBlue as an indirect measure of viability.

When averaged across all 96 drug combinations, the BSS for OVCAR8, HeyA8, and ES2 spheroids were within the additive range (i.e., −10 < BSS < 10; Figure 5). Interestingly, the BSS of TOV-21G spheroids was classified as antagonistic (i.e., BSS < −10). In adherent culture conditions, however, the BSS for all four cell lines were determined to be within the additive range (Supplemental Figure S3). Upon closer examination, however, distinct regions of synergy were observed within each heat map. In OVCAR8 and HeyA8 spheroids, these regions were found at medium to high concentrations of MRT68921 combined with low concentrations of afatinib. Within these regions, individual combination synergy scores ≥ 20 were identified, corresponding to a ≥20% response beyond what is mathematically expected [35,44]. In ES2 and TOV-21G spheroids, similar regions of potential synergy, albeit to a lesser level, were only seen at low concentrations of both MRT68921 and afatinib.

### 3.6. Combined MRT68921 and Afatinib Treatment Affects ULK1 Activity and Autophagy

Guided by these SynergyFinder studies, we pursued specific drug combinations that implied potential synergy for further analysis. Given the slightly stronger effects observed in OVCAR8 and HeyA8 spheroids, we used these lines for these experiments. Initial combination mapping in adherent and spheroid cultures of OVCAR8 and HeyA8 cells identified sub-IC_50_ concentration ranges that produced modest but reproducible regions of synergy. From these maps, we selected 4 µM MRT68921 + 0.125 µM afatinib and 0.25 µM MRT68921 + 0.125 µM afatinib for downstream experiments. Since 4 µM and 0.25 µM MRT68921 produced comparable reductions in p-Beclin-1 expression (Figure 1, Supplemental Figure S4), these concentrations were chosen to directly compare a lower and high concentration of MRT68921 when combined with afatinib.

**Figure 6:**
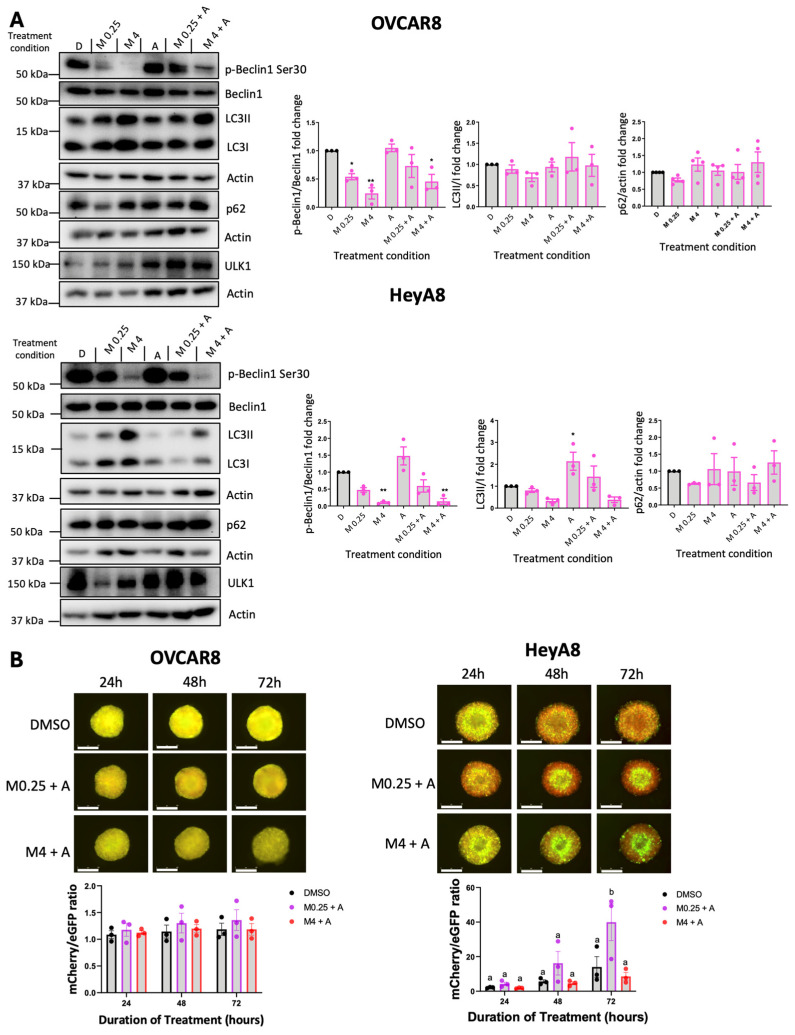
Combined MRT68921 and afatinib treatment modulates ULK1 activity and autophagy in EOC spheroids. (**A**) Representative immunoblots and densitometric analyses of OVCAR8 (n = 4) and HeyA8 (n = 3) spheroids treated either 0.25 μM (M0.25) or 4 μM (M4) of MRT68921 and 0.125 μM afatinib (A) for 24 h. Spheroids were seeded 24 h prior to drug treatment. Densitometric values are expressed as mean fold-change relative to DMSO-treated controls ± SEM. Black, and pink outlines denote DMSO-treated and afatinib-treated spheroids respectively. Statistical analysis was performed using one-way ANOVA followed by Tukey’s multiple comparisons test (* *p* < 0.05, ** *p* < 0.01). (**B**) Representative fluorescence images of OVCAR8-autoR and HeyA8-autoR spheroids treated with the indicated concentrations of MRT68921 and afatinib, beginning 24 h after seeding. Timepoints indicate the duration of treatment. Scale bars represent 400 μm. Autophagic flux was quantified based on the mCherry/eGFP fluorescence ratio and is presented as mean ± SEM (n = 3). Black, purple, and red outlines denote DMSO-, M0.25 + A-, and M4 +A-treated spheroids respectively. Statistical analysis was performed using two-way ANOVA followed by Tukey’s multiple comparisons test Tukey’s multiple comparisons test. Letter labels above bars indicate the presence (different letters) or absence (same letter) of significance at *p* < 0.05.

Given that afatinib and MRT68921 exert opposing effects on autophagy, we first examined the molecular consequences of their combination in OVCAR8 and HeyA8 spheroids using immunoblotting (Figure 6A). In OVCAR8 spheroids, treatment with 0.125 μM afatinib alone maintained p-Beclin1 levels comparable to the DMSO control. However, these levels were reduced when afatinib was combined with 4 μM MRT68921. As expected, MRT68921 alone at both tested concentrations decreased p-Beclin1 expression. Despite these effects on this ULK1-specific substrate, autophagy markers LC3-II/I and p62 remained largely unchanged across all conditions. In contrast, HeyA8 spheroids showed a reduction in p-Beclin1 following treatment with 4 μM MRT68921, either alone or in combination with afatinib. Interestingly, afatinib alone increased the LC3-II/I ratio in HeyA8 spheroids, but this effect was abolished when co-treated with MRT68921 at either concentration.

We next evaluated autophagic flux using OVCAR8- and HeyA8-autoR cell lines (Figure 6B). In OVCAR8-autoR spheroids, neither combination significantly altered autophagic flux, consistent with the lack of changes observed in LC3II/I and p62 expression (Figure 6A). However, in HeyA8 spheroids, 0.125 μM afatinib induced autophagic flux when combined with 0.25 μM MRT68921, as indicated by an increased red-to-green fluorescence ratio at 72 h. Notably, this effect was abolished when MRT68921 was increased to 4 μM, suggesting that higher concentrations of MRT68921 suppress afatinib-induced autophagy.

### 3.7. MRT68921 and Afatinib Combination Treatment Impairs Spheroid Viability and Reattachment

**Figure 7:**
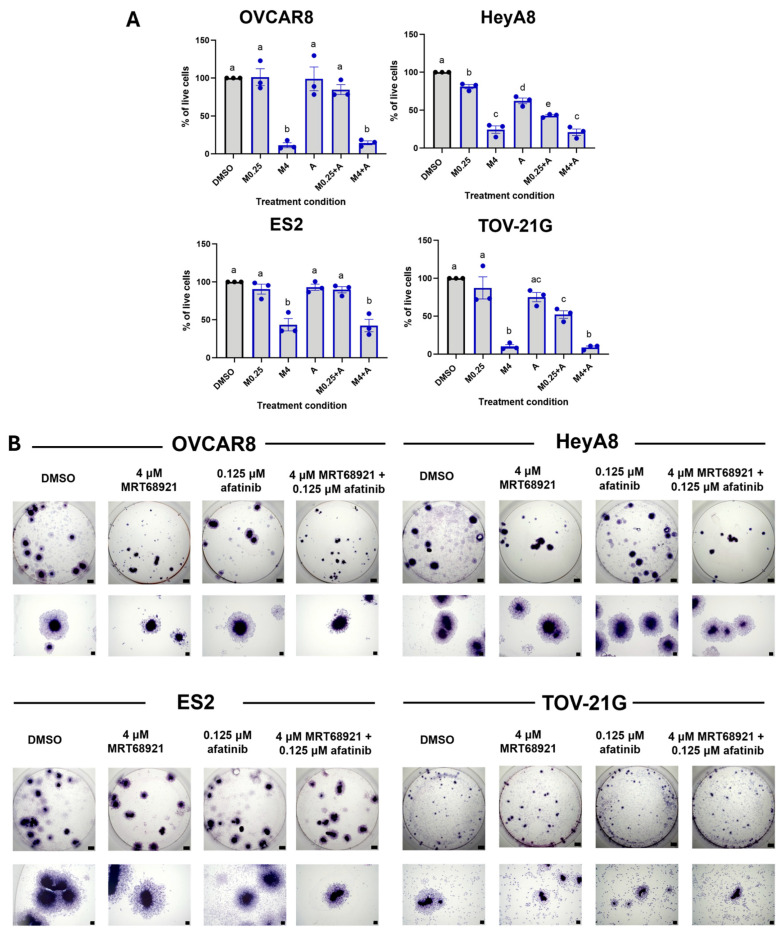
Combined MRT68921 and afatinib treatment impairs spheroid viability and reattachment. (**A**) Trypan Blue exclusion cell counting was performed on EOC spheroids following 72 h treatment with either 0.25 μM MRT68921 (M0.25) or 4 μM MRT68921 (M4) ± 0.125 μM afatinib (**A**). Data are presented as mean fold-change in percent viability normalized to DMSO control ± SEM (n = 3). Black, and blue outlines denote DMSO-treated and drug treated spheroids respectively. Statistical analysis was performed using one-way ANOVA followed by Tukey’s multiple comparisons test. Letter labels above bars indicate the presence (different letters) or absence (same letter) of significance at *p* < 0.05. (**B**) Representative images of Hema3-stained spheroids following a 72 h drug treatment and subsequent transfer to 24-well tissue culture plastic dishes for reattachment. Individual well and high-power images show qualitative differences in spheroid morphology and reattachment capacity across treatment conditions (n = 4). Scale bars represent 500 μm.

We next expanded our analysis of the combined effect of MRT68921 and afatinib on spheroid cell viability. HeyA8 and OVCAR8 spheroids exhibited a generally better response to the combination treatments as visualized by our Synergy Finder assays as compared with ES2 and TOV-21G spheroids (Figure 5). However, these latter two cell lines exhibited a small but consistent area of moderate synergy at lower concentrations of both MRT68921 and afatinib. To maintain consistency in the following experiments and allow for direct comparison of treatment responses among histologically distinct models, we selected the same representative concentration pairs of low concentration MRT68921 (0.25 μM) or high concentration MRT68921 (4 μM MRT68921) ± low concentration afatinib (0.125 μM). These concentrations were selected to fall within or adjacent to at least one dose combination observed among the four cell lines. Accordingly, OVCAR8, HeyA8, ES2, and TOV-21G spheroids were treated under these conditions, and direct cell viability was assessed after 72 h (Figure 7A).

Trypan Blue Exclusion cell counting revealed reduced viability across all spheroid models following treatment with MRT68921 and afatinib. In HeyA8 and TOV-21G spheroids, the combination of 0.25 μM MRT68921 and 0.125 μM afatinib resulted in greater viability reduction compared to either agent alone, consistent with Synergy Finder predictions. Notably, treatment with 4 μM MRT68921, either alone or in combination with afatinib, produced the most pronounced effects, reducing spheroid viability by over 70% in OVCAR8, HeyA8, and TOV-21G cell lines, and by approximately 60% in ES2 spheroids.

To further evaluate the functional consequences of combined MRT68921 and afatinib treatment, we performed spheroid reattachment assays, which offer insight into mechanisms controlling the transition from dormancy to a proliferative state [8,12,45]. Following 72 h of treatment, spheroids were transferred to adherent conditions and stained with Hema3 to visualize reattachment and cellular outgrowth (Figure 7B). Consistent with Trypan Blue viability data, 4 μM MRT68921 alone or in combination with 0.125 μM afatinib markedly impaired spheroid reattachment across all cell lines. This suggests that ULK1 inhibition alone compromises spheroid cell viability and, consequently, their ability to reattach to a solid substratum.

Although Synergy Finder analysis identified localized regions of synergy between MRT68921 and afatinib at select concentration ranges, ULK1 inhibition via MRT68921 alone produced the most consistent reduction in spheroid viability and reattachment across all cell lines tested.

### 3.8. MRT68921 and Afatinib Combination Treatment Impairs Ascites-Derived Organoid Viability

**Figure 8:**
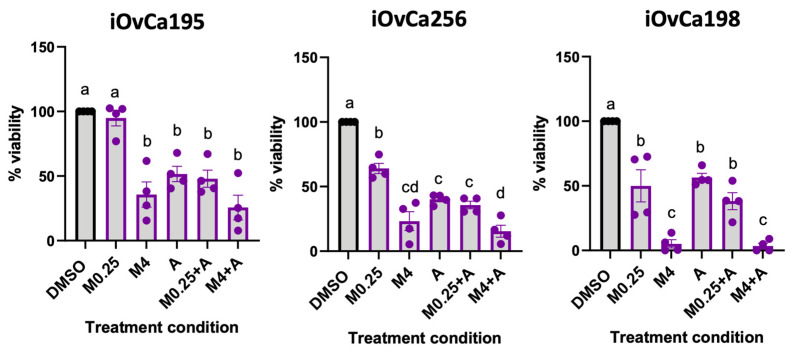
Effect of combined MRT68921 and afatinib treatment on patient ascites-derived organoids. Patient ascites-derived organoids were seeded and allowed to mature for 72 h prior to 72 h treatment with either 0.25 μM MRT68921 (M0.25) or 4 μM MRT68921 (M4) ± 0.125 μM afatinib (A). Cell viability was assessed using the alamarBlue assay and expressed as percent viability relative to DMSO-treated controls ± SEM (n = 4). Black, and purple outlines denote DMSO-treated and drug treated organoids respectively. Statistical analysis was performed using one-way ANOVA followed by Tukey’s multiple comparisons test. Letter labels above bars indicate the presence (different letters) or absence (same letter) of significance at *p* < 0.05.

To extend our findings from spheroid models of EOC metastasis, we next evaluated combined MRT68921 and afatinib treatment effects in patient-derived ascites organoids. Organoids are increasingly recognized as powerful in vitro tools for therapeutic testing due to their ability to recapitulate patient tumour heterogeneity and architecture [46,47,48,49]. We leveraged ascites-derived HGSOC organoids generated from our iOvCa cell lines, which represent samples from patients with diverse treatment histories, molecular profiles, and therapeutic responses [50]. Organoids were established for 7 days, then treated for 72 h, and viability was assessed using the alamarBlue assay (Figure 8).

iOvCa195 and iOvCa256 organoids exhibited similar sensitivity across all treatment conditions, except for 0.25 μM MRT68921, which did not reduce the viability of iOvCa195 organoids. In contrast, iOvCa198 organoids demonstrated a pronounced response to MRT68921, with 4 μM MRT68921 alone or in combination with 0.125 μM afatinib reducing viability by over 80%. Notably, afatinib did not further enhance the effects of 4 μM MRT68921, likely due to the strong response elicited by MRT68921 alone.

Interestingly, all iOvCa organoids demonstrated greater sensitivity to MRT68921 and afatinib compared to established spheroid models, with viability reduced by at least 50% in many treatment conditions.

## 4. Discussion

EOC remains a highly lethal malignancy due to its propensity for peritoneal dissemination, late-stage detection, and chemoresistance [51,52]. Autophagy has emerged as a critical survival mechanism in EOC, particularly in response to metabolic stress and therapy-induced cytotoxicity [14,19,53]. Previously, we demonstrated that EOC spheroids induce autophagy in an ULK1-dependent manner and that autophagy supports spheroid cell survival, making it a compelling therapeutic target [14,16]. We were the first to investigate ULK1-targeted inhibition in EOC using MRT68921 [16]. Here, we expand upon those findings by showing that MRT68921 effectively disrupts autophagy and reduces both spheroid and organoid viability in HGSOC and OCCC cell lines. Additionally, we establish that afatinib, a widely studied TKI, induces autophagy in EOC cells and spheroids, prompting us to evaluate its potential in combination with MRT68921. While MRT68921 and afatinib exhibited synergy at select concentrations, MRT68921 alone had the most pronounced effects on reducing cell viability, spheroid reattachment, and organoid survival. These findings enhance our understanding of ULK1-targeting strategies in EOC and highlight their translational potential.

Our previous work demonstrated the effectiveness of MRT68921 exclusively in HGSOC [16]. Recognizing the heterogeneity across EOC histotypes, we expanded these findings by including additional HGSOC cell lines and OCCC cell lines, given the latter’s poor response to conventional combination chemotherapy [4,5]. Here, we showed that MRT68921 effectively inhibits ULK1 activity in both HGSOC and OCCC, as evidenced by near-complete loss of p-Beclin1 Ser30, a direct ULK1 substrate [36,37,38]. Given the canonical role of ULK1 in autophagy initiation, it is unsurprising that ULK1 inhibition reduced autophagic flux in HeyA8 spheroids. However, OVCAR8 spheroids exhibited limited changes in LC3-II/I ratio, p62 expression, and mCherry/eGFP ratio following MRT68921 treatment, suggesting heterogeneity in autophagy regulation among EOC cell lines. This is consistent with our work demonstrating that HeyA8 and OVCAR8 spheroids display distinct spatial patterns of autophagy activation, with HeyA8 spheroids exhibiting rapid and robust autophagy activation within their core (Borrelli & Shepherd, unpublished results). These differences were particularly evident when fluorescence was analyzed temporally using our newly established SPoRTS analysis pipeline [34]. Further investigation into how ULK1 inhibition affects spatially distinct autophagy activation patterns within spheroids, beyond what is captured by bulk fluorescence signal analysis, may offer valuable insights into mechanisms controlling autophagy in EOC and heterogeneity among different histotypes.

This heterogeneity is further underscored by the variable sensitivity of EOC cell lines to MRT68921. Although IC_50_ values of cell lines in monolayer culture were within a narrow range, no trend was observed regarding cell line sensitivity to MRT68921 during spheroid formation (early spheroids) or in maintaining spheroid viability (late spheroids). Interestingly, all cell lines, except for HeyA8, exhibited greater sensitivity to MRT68921 under adherent conditions compared to suspension culture, suggesting a differential reliance on ULK1 between adherent and suspension cell states. This is consistent with our previous findings, which showed increased ULK1 protein expression in HGSOC spheroids relative to monolayer culture [16]. The reduced sensitivity of spheroids to MRT68921 may also reflect their inherent 3D architecture, which can create nutrient and oxygen gradients [54,55], potentially limiting MRT68921 penetration into the spheroid core. Indeed, prior studies comparing IC_50_ values between adherent and spheroid cultures have demonstrated that cytotoxic agents often exhibit reduced efficacy in spheroids due to slow diffusion rates [56]. Taken together, these findings highlight the biological complexity of 3D spheroid models and reinforce the importance of evaluating drug efficacy in systems that more closely recapitulate in vivo tumour conditions, albeit without vascularization [49].

TKIs, including afatinib, induce autophagy in various cancers, where its activation is implicated as a resistance mechanism that sustains cell survival under therapeutic stress [30,31,57,58]. Here, we demonstrate for the first time that afatinib induces autophagy in both HGSOC and OCCC cell lines, as evidenced by an increased LC3-II/I ratio and enhanced autophagic flux in OVCAR8- and HeyA8-autoR spheroids. Although we did not directly evaluate autophagy as an afatinib-induced resistance mechanism in EOC cells, future studies could investigate whether this may be implicated in EOC, particularly in spheroid models of metastasis.

Previous studies have shown that combining afatinib with autophagy inhibitors enhances its anti-cancer effects in NSCLC, HNSCC, and neuroblastoma [30,31,58]. Additionally, our group has previously demonstrated that combining AKT inhibition with autophagy blockade using chloroquine or Spautin-1 leads to synergistic reductions in EOC cell viability [42]. This aligns with a growing clinical interest in ULK1-targeted therapies, as highlighted by the development of the ULK1/2 inhibitor DCC-3116 (inlexisertib) [59]. In fact, DCC-3116 is currently being evaluated in a phase I/II trial in combination with MEK inhibitors for patients with solid tumours bearing RAS/MAPK pathway mutations.

Building on this rationale, we evaluated the combination of afatinib with MRT68921 using EOC models, and our Synergy Finder analysis identified distinct regions of potential synergy. In OVCAR8 and HeyA8 cell lines, these regions were at moderate to high concentrations of combined with low afatinib concentrations. In ES2 and TOV-21G cell lines, these regions of somewhat reduced synergy were identified at lower concentrations of both drugs. It should be noted, however, that the overall synergy score across all concentration combinations in adherent and spheroid culture conditions predicted an average additive effect, with the exception of TOV-21G spheroids which was antagonistic. Interestingly, among these four cell lines, adherent TOV-21G cells were most sensitive to MRT68921 alone (IC_50_ value of 0.760 μM) as compared with ES2 (2.384 μM), OVCAR8 (4.192 μM), and HeyA8 (4.242 μM). Our additional cell viability and functional assays demonstrated that MRT68921 alone was effective to block these activities, perhaps due to a ceiling effect of ULK1 inhibition for these specific assays. One possible explanation for this observation may be the high dependence of EOC spheroids on ULK1 for survival, which may limit the additional benefit of TKI-induced autophagy modulation [14,15,16]. We speculate that TOV-21G cells are particularly vulnerable to the ceiling effect due to their increased sensitivity to MRT68921.

We kept combination concentrations of MRT68921 and afatinib consistent throughout experiments and among cell lines to facilitate their direct comparison across HGSOC and OCCC cell histotypes. While this represented a simple framework for direct comparison, it may represent a limitation of our study. Our experiments following Synergy Finder analysis revealed cell line- and patient-specific differences in viability and spheroid attachment in response to combination treatment, thus highlighting the known heterogeneity of EOC. Accordingly, regions of potential synergy indicated by Synergy Finder experiments of specific cell lines using a single viability readout may not yield optimal drug combination ratios for individual EOC samples.

Beyond its effects on spheroid viability, ULK1 inhibition via MRT68921 induced notable morphological changes in reattached spheroids. Spheroids treated with 4 µM MRT68921, either alone or in combination with afatinib, were smaller and exhibited reduced reattachment, particularly in OVCAR8 and HeyA8 cell lines. These findings suggest that ULK1 and autophagy play critical roles in spheroid plasticity and peritoneal metastasis, a hallmark of late-stage EOC. Targeting ULK1 may therefore represent a potential anti-metastatic strategy, warranting further investigation using additional functional assays such as mesothelial clearance or cell migration assays [60,61] to directly assess the ability of MRT68921 to impair metastatic potential. Supporting this notion, we have new evidence that ULK1 knockout in EOC cell lines impairs spheroid ability to displace a mesothelial monolayer [62], further implicating ULK1 as a key regulator of motility and metastatic capacity in EOC.

One of the most striking findings of this study was the heightened sensitivity of patient ascites-derived HGSOC organoids to MRT68921. These models better recapitulate tumour-extracellular matrix (ECM) interactions and retain the phenotypic properties of patient tumours [46,47,48,49]. Notably, MRT68921 reduced viability by over 80% in some organoids, reinforcing the therapeutic potential of ULK1 inhibition in EOC. At the time of study, our biobank primarily consists of samples that represent HGSOC. However, ongoing biobanking efforts are actively expanding to include OCCC and other EOC histotypes. The application of these additional patient-derived organoid samples will enable future studies to evaluate whether observed sensitivity to ULK1 inhibition extends across histologically and genetically diverse models of EOC. Identifying trends in ULK1 inhibitor sensitivity, related to genetic profiling data or specific ULK1 biomarker assays (e.g., p-Beclin1 Ser30) that could inform clinical decision-making in the future. A comprehensive approach integrating preclinical models and validating additional ULK1-targeted agents will be essential to elucidate the mechanisms underlying ULK1 dependence and to evaluate its therapeutic potential in EOC.

## 5. Conclusions

Overall, our findings underscore ULK1 as a promising therapeutic target in EOC. We demonstrate that MRT68921 effectively inhibits ULK1 activity and disrupts autophagy in both HGSOC and OCCC histotypes. When combined with afatinib, we observed primarily additive or antagonistic effects, but with discrete regions of potential synergy. However, organoid and spheroid reattachment models specific to EOC revealed that the predominant cytotoxic effects were driven by ULK1 inhibition alone. Notably, patient-derived ascites organoids displayed marked heterogeneity in treatment response reflecting the biological diversity of EOC. These findings collectively support the therapeutic potential of ULK1 inhibition while emphasizing the need for histotype-specific optimization of combination dosing strategies in future studies.

## Figures and Tables

**Table 1:** Advanced DMEM/F-12 media additives for organoid culture.

Additive	Concentration
B-27^TM^ (#17504044) ^1^	1×
Forskolin (F6886) ^2^	10 μM
GlutaMAX^TM^ (#35050061) ^1^	1×
HEPES (#600-032 LG) ^3^	10 mM
Human Epidermal Growth Factor (#AF-100-15) ^4^	20 ng/mL
Human Fibroblast Growth Factor-10 (#100-26) ^4^	100 ng/mL
Nicotinamide (N3376) ^2^	1 mM
N-Acetyl-L-cysteine (A7250) ^2^	1.25 mM
Recombinant Human Noggin Protein (#6057-NG) ^5^	100 ng/mL
ROCK Inhibitor (Y-27623) (SXM075) ^2^	10 μM

^1^ Thermo Fisher Scientific, Waltham, MA, USA ^2^ MilliporeSigma, Burlington, MA, USA ^3^ Wisent, Saint-Jean-Baptiste, QC, Canada ^4^ Preprotech Inc., Cranbury, NJ, USA ^5^ R & D Systems, Minneapolis, MN, USA.

## Data Availability

The original contributions presented in this study are included in the article/Supplementary Material. Further inquiries can be directed to the corresponding author. The raw data supporting the conclusions of this article will be made available by the authors on request.

## Supplementary Materials

The following supporting information can be downloaded at: https://www.mdpi.com/article/10.3390/cancers18020307/s1, Figure S1: MRT68921 inhibits ULK1 activity for at least 96 h in EOC culture models; Table S1: MRT68921 IC_50_ values of EOC cell lines in adherent culture; Figure S2: Afatinib alters AKT signalling in EOC cell lines; Figure S3: Synergy Finder analysis of MRT68921 and afatinib combination treatment in adherent culture. Figure S4: MRT68921 inhibits ULK1 activity in EOC cell lines.

## Abbreviations

The following abbreviations are used in this manuscript:

ADH	Adherent
AIC	Autophagy initiation complex
AKT	Protein kinase B
AMPK	AMP-activated protein kinase
ATCC	American Type Culture Collection
ATG101	Autophagy-related protein 101
ATG13	Autophagy-related protein 13 homolog
autoR	mCherry-eGFP-LC3B reporter
BSA	Bovine serum albumin
BSS	Bliss synergy score
CAMKK2	Calcium/calmodulin-dependent protein kinase kinase 2
DMEM	Dulbecco’s modified Eagle medium
ECM	Extracellular matrix
EGFR	Epidermal growth factor receptor
EOC	Epithelial ovarian cancer
FACS	Fluorescence-activated cell sorting
FBS	Fetal bovine serum
FIP200	FAK family-interacting protein of 200 kDa
HGSOC	High grade serous ovarian cancer
HNSCC	Head and neck squamous cell carcinoma
HRP	Horseradish peroxidase
IC_50_	Half-maximal inhibitory concentration
LC3	Microtubule-associated protein 1 light chain 3
mTORC1	Mammalian target of rapamycin complex 1
NSCLC	Non-small cell lung cancer
OCCC	Ovarian clear cell carcinoma
p62	Sequestosome 1
PBS	Phosphate-buffered saline
PDAC	Pancreatic ductal adenocarcinoma
PMSF	Phenylmethylsulfonyl fluoride
PVDF	Polyvinylidene fluoride
RIPA	Radioimmunoprecipitation assay
RPMI	Roswell Park Memorial Institute medium
SDS	Sodium dodecyl sulfate
SPH	Spheroid
SPoRTS	Spatial Profiling of Ratiometric Trends in Spheroids
STR	Short tandem repeat
TBST	Tris-buffered saline-Tween 20
TCGA	Centre for Applied Genomics
TKI	Tyrosine kinase inhibitor
ULA	Ultra-low attachment
ULK1	Unc-51-like autophagy activating kinase 1
ULK2	Unc-51-like autophagy activating kinase 2
