# Supplementary material for: Evaluating the Therapeutic Potential of MRT68921 and Afatinib in Three-Dimensional Models of Epithelial Ovarian Cancer
Source: Cancers (Basel). 2026 Jan 19;18(2):307. doi: 10.3390/cancers18020307 (PMC12839298; doi:10.3390/cancers18020307)
Supplement: Supplementary file 1 [file cancers-18-00307-s001.zip › cancers-4066361-supplementary.pdf]

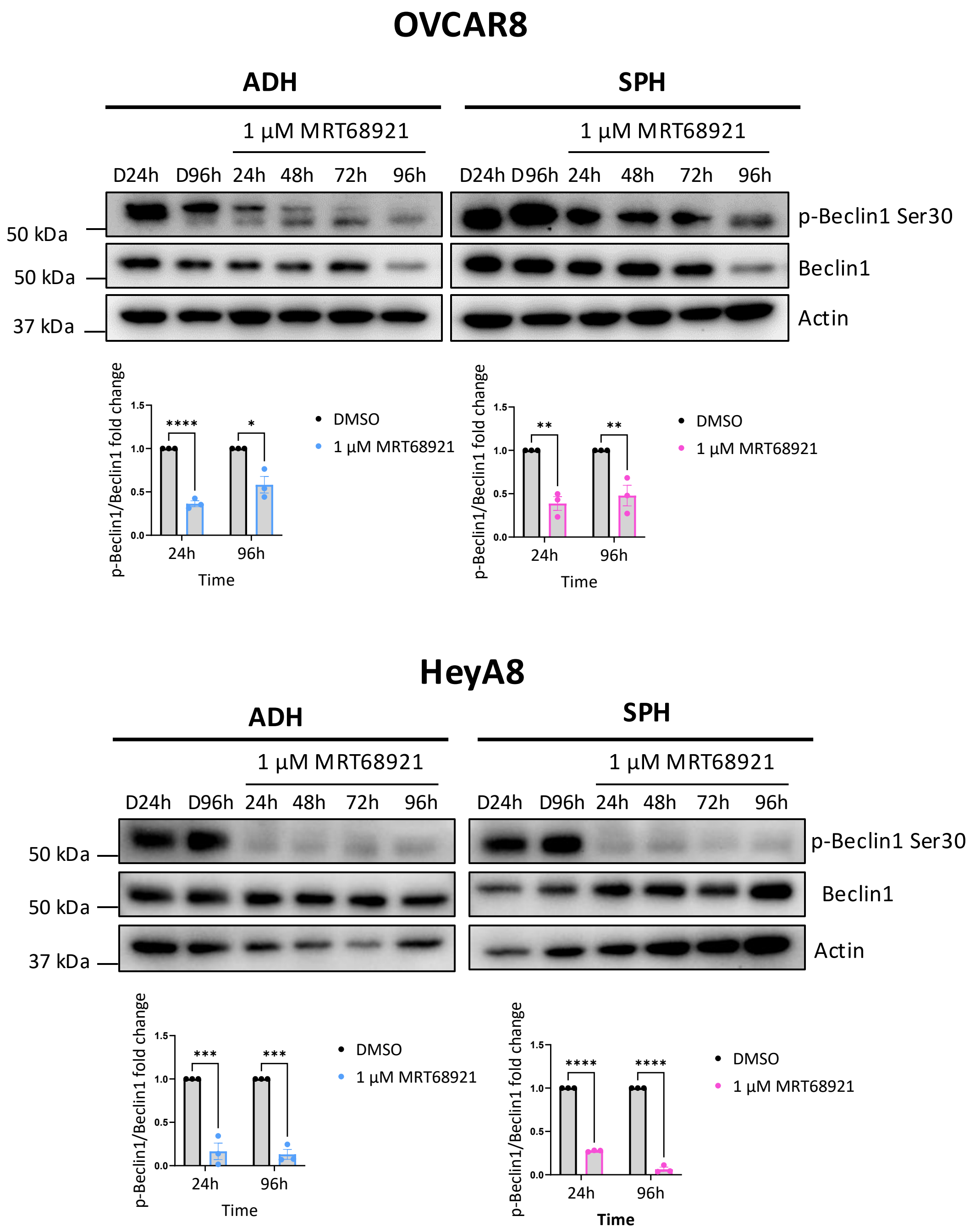

Supplemental Figure S1. MRT68921 inhibits ULK1 activity for at least 96 hours in EOC culture models. Representative immunoblots and corresponding densitometric analyses of OVCAR8 (n = 3) and HeyA8 (n = 3) cells cultured under adherent (ADH) and spheroid (SPH) conditions. Cells were seeded 24 hours prior to treatment with 1 µM MRT68921 and harvested after 24, 48, 72, or 96 hours of drug exposure. Densitometric data represent mean fold change at 24 and 96 hours, normalized to their respective DMSO controls (D24h and D96h) ± SEM. Statistical significance was determined using unpaired t-tests comparing treated versus control samples at each time point (*$p < 0.05$, **$p < 0.01$, ***$p < 0.001$, ****$p < 0.0001$).

Supplemental Table S1. MRT68921 IC_{50} values of EOC cell lines in adherent culture.

Cell line	Histotype	IC_{50} (μM) ± SEM
OVCAR8	HGSOC	4.192 ± 0.297
HeyA8	Poorly differentiated	4.242 ± 0.081
COV318	HGSOC	3.038 ± 0.524
COV362	HGSOC	2.325 ± 0.126
OVCAR3	HGSOC	3.004 ± 0.085
OVCAR4	HGSOC	2.649 ± 0.061
ES2	OCCC	2.384 ± 0.125
TOV-21G	OCCC	0.760 ± 0.126
OV207	OCCC	3.147 ± 0.217
105C	OCCC	1.285 ± 0.161

Note: 12-point dose-response curves were generated and IC_{50} values were determined using the non-linear regression (curve fit) analysis tool in GraphPad Prism 9.5.1.

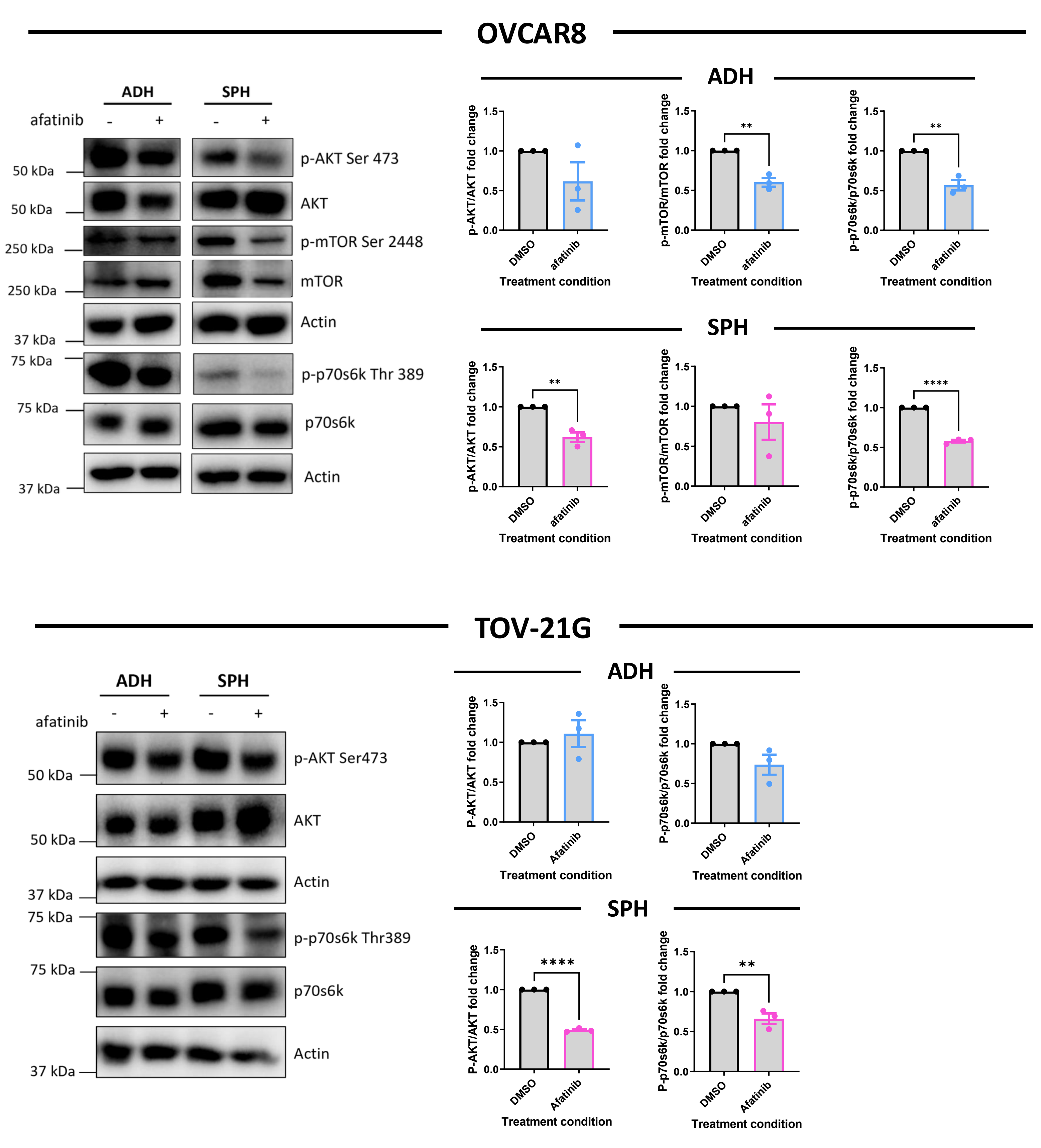

Supplemental Figure S2. Afatinib alters AKT signaling in EOC cell lines. Representative immunoblots and densitometric analyses of OVCAR8 and TOV-21G cells treated with 4 μM afatinib for 24 hours under adherent (ADH) and spheroid (SPH) culture conditions. Cells were seeded and allowed to adhere for 24 hours prior to treatment. Densitometric values represent mean fold change relative to DMSO-treated controls ± SEM (*n=3*). Statistical analysis was performed using unpaired t-tests (**p < 0.01, ****p < 0.0001).

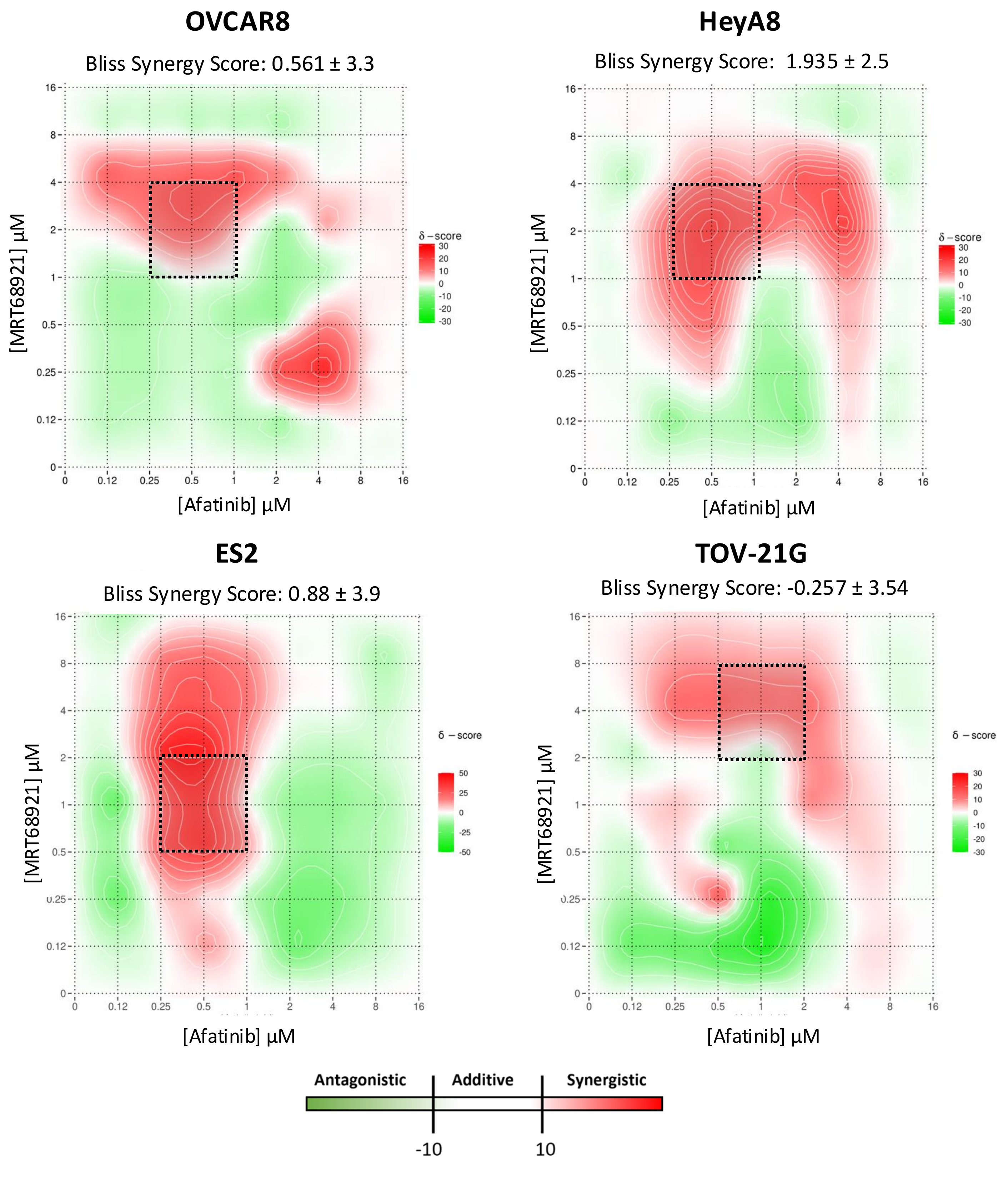

Supplemental Figure S3. Synergy Finder analysis of MRT68921 and afatinib combination treatment in adherent culture. Bliss synergy scores (BSS) are presented as mean ± SEM (n = 3). Heat maps display the average synergy scores from biological replicates, where red indicates synergistic interaction (BSS > 10), white represents additive effects (−10 < BSS < 10), and green indicates antagonism (BSS < −10). Regions enclosed by dashed-lined boxes represent areas with the greatest degree of synergy. All cells were seeded 24 hours prior to 72-hour combination treatment with afatinib and MRT68921, using a matrix of concentrations ranging from 0 to 16 µM. Cell viability was assessed using alamarBlue as an indirect measure of viability.

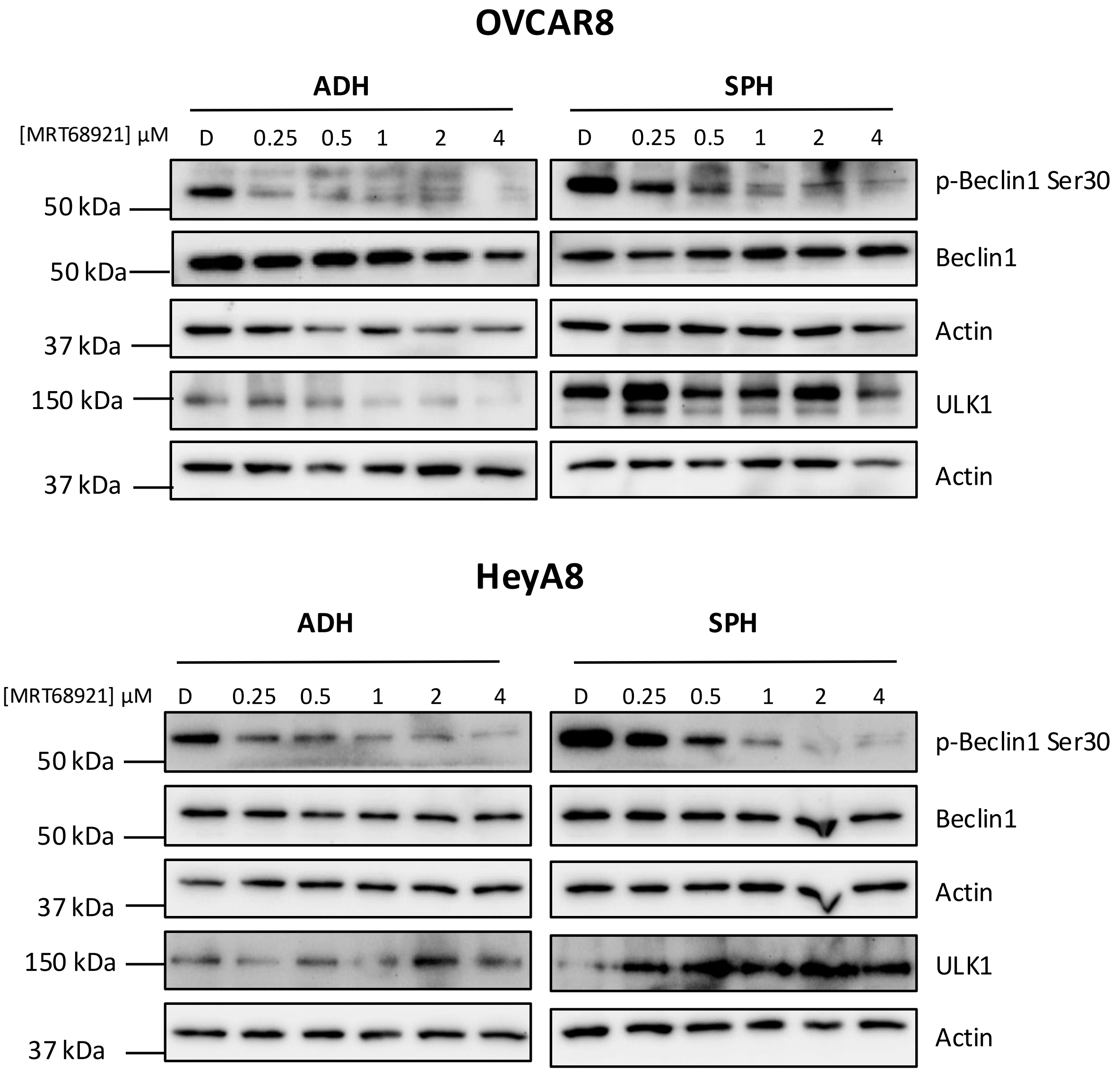

Supplemental Figure S4. MRT68921 inhibits ULK1 activity in EOC cell lines. Representative immunoblots of OVCAR8 and HeyA8 cells treated with the indicated concentrations of MRT68921 under adherent (ADH) and spheroid (SPH) culture conditions. Cells were seeded 24 hours prior to a 24-hour treatment with MRT68921.
